# An economic evaluation of maxillary implant overdentures based on six vs. four implants

**DOI:** 10.1186/1472-6831-14-105

**Published:** 2014-08-18

**Authors:** Stefan Listl, Leonhard Fischer, Nikolaos Nikitas Giannakopoulos

**Affiliations:** 1 Department of Conservative Dentistry, Heidelberg University, Im Neuenheimer Feld 400, 69120 Heidelberg, Germany; 2 Max Planck Institute for Social Law and Social Policy, Munich Center for the Economics of Aging, Munich, Germany; 3 Department of Conservative Dentistry, Heidelberg University, Heidelberg, Germany; 4 Department of Prosthodontics, Heidelberg University, Heidelberg, Germany

**Keywords:** Cost-effectiveness analysis, Implants, Dentures, Maxilla

## Abstract

**Background:**

The purpose of the present study was to assess the value for money achieved by bar-retained implant overdentures based on six implants compared with four implants as treatment alternatives for the edentulous maxilla.

**Methods:**

A Markov decision tree model was constructed and populated with parameter estimates for implant and denture failure as well as patient-centred health outcomes as available from recent literature. The decision scenario was modelled within a ten year time horizon and relied on cost reimbursement regulations of the German health care system. The cost-effectiveness threshold was identified above which the six-implant solution is preferable over the four-implant solution. Uncertainties regarding input parameters were incorporated via one-way and probabilistic sensitivity analysis based on Monte-Carlo simulation.

**Results:**

Within a base case scenario of average treatment complexity, the cost-effectiveness threshold was identified to be 17,564 € per year of denture satisfaction gained above of which the alternative with six implants is preferable over treatment including four implants. Sensitivity analysis yielded that, depending on the specification of model input parameters such as patients’ denture satisfaction, the respective cost-effectiveness threshold varies substantially.

**Conclusions:**

The results of the present study suggest that bar-retained maxillary overdentures based on six implants provide better patient satisfaction than bar-retained overdentures based on four implants but are considerably more expensive. Final judgements about value for money require more comprehensive clinical evidence including patient-centred health outcomes.

## Background

Implant-retained overdentures have become an important treatment option of modern dentistry. Such treatment presents the prospect of high levels of oral health related quality of life and is particularly important in times of population aging as edentulousness rates continue to be relevantly high [1]. For mandibular implant-based overdentures, current consensus is that patients’ satisfaction and quality of life is significantly greater for implant-supported overdentures than for conventional dentures and that a two-implant mandibular overdenture should be the minimum treatment standard for most patients [2]. Not least, the availability of evidence already facilitated an assessment of the cost-effectiveness of implant-retained mandibular overdentures [3].

Yet comparably little evidence and consensus seem to exist with respect to implant-based overdentures for treatment of the edentulous maxilla. Maxillary overdentures have however been considered a relevant treatment alternative, particularly when retention and stability of conventional dentures is dissatisfactory [4]. It was suggested that a number of four implants would be the minimum to support a maxillary overdenture and six implants would provide additional clinical advantages [5]. In a relevant meta-analysis, most evidence on the clinical performance of maxillary overdentures was identified to originate from studies examining either six or four implants connected with a bar [6]. Recently, moreover, some evidence became available about patient-reported outcomes of maxillary implant-supported overdentures [7-9]. It yet remains unclear whether the value gained by six instead of four implants within bar-retained implant overdentures outweighs the potentially higher costs.

To our knowledge, the cost-effectiveness of maxillary overdentures based on six or four implants has never been investigated before. Therefore, the purpose of the present study was to assess, on basis of currently available evidence, the value for money achieved by bar-retained implant overdentures with six implants compared with four implants as treatment alternatives for the edentulous maxilla.

## Methods

**Figure 1:**
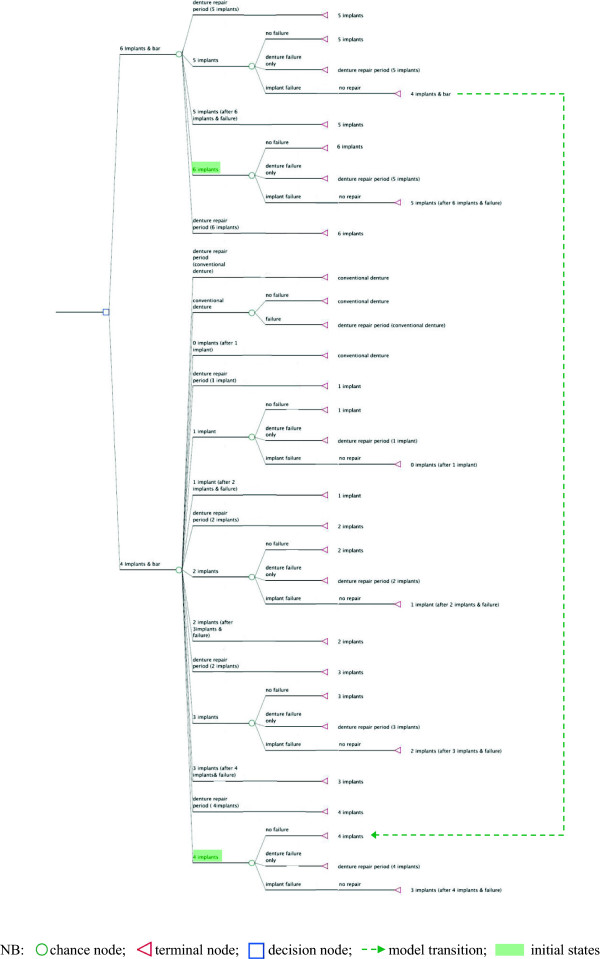
The Markov decision tree.

The present study is based on secondary analysis of previously published material. Therefore, approval by an ethics committee was not required. The perspective considered in this paper is that of a decision maker who seeks optimization from a societal perspective [10]. Because social health insurance often does not cover the expenses of maxillary implant-supported overdentures, this perspective corresponds closely to the perspective of the individual patient who wants to understand whether investment in a six implant overdenture is preferable to investment in a four-implant overdenture. As such, the knowledge generated through the present analysis is likewise relevant for clinical practitioners who want to inform their patients transparently about suitable treatment alternatives. We assume that the decision making process is taking place in Germany and model the data within a Markov decision tree (Figure 1). Markov models are a health economic technique to depict probabilities and time durations for cycles in which individuals remain in the same or move on to different health states. Thereby, health states are assigned health outcomes and costs, resulting in characteristic cost-outcome profiles in response to treatment decisions [11].

### Model input parameters

#### Implant and denture failure

A literature search via PubMed/Medline (April 9th 2014) found six meta-analyses for the search terms "maxillary overdenture", "upper jaw overdenture", "implant overdenture, maxilla", "maxillary over-denture", "upper jaw over-denture", and "implant over-denture, maxilla" [5,6,12-15]. From these six meta-analyses, five did not facilitate comparisons of various numbers of implants for maxillary overdentures and were thus excluded from the present analysis [5,12-15]. The remaining meta-analysis by Slot et al. [6] was the only one which provided comparisons of various numbers of implants and respective information on implant and denture failure. The information from this study was therefore included for the purpose of our investigation.

Slot et al. [6] provide information on bar-retained maxillary implant overdentures after a mean observation period of at least one year and report respective failure rates for dentures combined with either six or four implants. Based on this information, we modelled the treatment decision of restoring the edentulous maxilla by means of a six implant or a four implant based bar-retained overdenture and defined the respective treatment alternatives as entry points into our Markov model (Figure 1). For further parameterization of our model we assumed that, throughout annual cycles of the Markov model, implants and overdentures could remain either sound, or an implant failure could occur, or a denture failure could occur. Thereby, it was assumed that failure rates occur independent of each other and that implants are not replaced once lost. The available meta-evidence provides information for failure rates in relation to six or four implants only [6]. Therefore, failure rates for other implant numbers were linearly interpolated. For failure of dentures without implants, an additional triangular distribution was assumed. Implant and denture failure parameters incorporated in the present study are summarized in Table 1.

**Table 1:** Annual failure rates and health outcome parameters used in the analysis (base case scenario)

	**Distribution**	**Distribution parameters**	**Data Source**
*Implant failure*			
6 implants	Triangular	Mode 0.018 (min-max 0.014-0.023)	[6]
5 implants	Triangular	Mode 0.0275 (min-max 0.022-0.0345)	Interpolated from [6]
4 implants	Triangular	Mode 0.037 (min-max 0.030-0.046)	[6]
3 implants	Triangular	Mode 0.0465 (min-max 0.038-0.0575)	Interpolated from [6]
2 implants	Triangular	Mode 0.056 (min-max 0.046-0.069)	Interpolated from [6]
1 implant	Triangular	Mode 0.0655 (min-max 0.054-0.0805)	Interpolated from [6]
*Denture failure*			
6 implants	Triangular	Mode 0.026 (min-max 0.015-0.044)	[6]
5 implants	Triangular	Mode 0.0305 (min-max 0.0185-0.0565)	Interpolated from [6]
4 implants	Triangular	Mode 0.035(min-max 0.022-0.069)	[6]
3 implants	Triangular	Mode 0.0395 (min-max 0.0255-0.0815)	Interpolated from [6]
2 implants	Triangular	Mode 0.044 (min-max 0.029-0.094)	Interpolated from [6]
1 implant	Triangular	Mode 0.0485 (min-max 0.0325-0.1065)	Interpolated from [6]
no implants	Triangular	Mode 0.05 (min-max 0.01-0.10)	Assumption
*Patient satisfaction* [1: full satisfaction; 0: no satisfaction]			
6 implants	Point estimate	0.89	[8]
5 implants	Point estimate	0.89	Interpolated from [8]
4 implants	Point estimate	0.89	[8]
3 implants	Point estimate	0.865	Interpolated from [7,8]
2 implants	Point estimate	0.84	[7]
1 implant	Point estimate	0.735	Interpolated from [7]
no implants	Point estimate	0.63	[7]
Repair period adjustment factor [1: full satisfaction; 0: no satisfaction]	Triangular	Mode 0.9 (min-max 0.8-0.99)	Assumption

#### Patient satisfaction

As relevant health outcome parameter, we populated the Markov decision tree model (Figure 1) with information about patient satisfaction with overdentures as available from recent literature. For denture satisfaction in relation to six and four implants, we relied on overall patient satisfaction with implant-retained maxillary overdentures as reported by Slot et al. [8]. Note that this study reports outcomes for implants located in the posterior maxilla. This seems justifiable given that a further study reported similar outcomes for the anterior maxilla and confirmed that patient satisfaction with dentures does not differ significantly between four and six implants [9]. For patients’ satisfaction in relation to two or no implants, we relied on recently reported findings from Zembic & Wismeijer [7] on general satisfaction with fitted conventional and two-implant-retained maxillary dentures. Note that the results from Slot et al. and from Zembic & Wismeijer were reported on different scales and on basis of different sample sizes [7,8]. Therefore, the respective parameter values were included as point estimates and rescaled such that a value of 1 always indicates perfect satisfaction and zero indicates total dissatisfaction. In order to model denture satisfaction associated with five, three, and one implant(s), the respective values were linearly interpolated. Moreover, compromised patient satisfaction due to temporary denture non-functionality throughout repair periods was incorporated via a triangularly distributed satisfaction adjustment parameter. Patient satisfaction parameters incorporated in the present study are summarized in Table 1.

#### Costs

As the decision making process was assumed to take place in Germany, calculation of cost parameters relied on the relevant reimbursement regulations within the German health care system. Specifically, the dental personnel costs associated with provision of new maxillary implant overdentures and care after implant loss or denture failure were calculated in accordance with the "Gebührenordnung für Zahnärzte" [16,17]. The relevant regulations allow for consideration of various treatment complexity and required treatment times for several treatment elements and, accordingly, different extents of treatment cost. In the present study, such treatment time variation is implemented via a cost multiplication factor which is assumed to take on values of 1.0 (low treatment complexity), 2.3 (average treatment complexity), or 3.5 (high treatment complexity); these multiplication factors are commonly referred to for cost calculations on basis of the "Gebührenordnung für Zahnärzte" [16]. Cost calculations were based on information of an online cost calculator ( http://www.synadoc.de) [18]. Bar constructions and further anchoring elements were assumed to be manufactured out of predominantly base alloys. Laboratory and material costs for denture repair were calculated according to the standards of the Heidelberg University, Department of Prosthodontics dental lab. Cost parameters incorporated in the present study are summarized in Table 2.

**Table 2:** Cost parameters used in the analysis [in €]

	**Factor 1.0**	**Factor 2.3**	**Factor 3.5**	**Data source**
*Dentist labor costs*				
Six-implant over-denture (new)	1,472.67	2,990.86	4,392.27	[16-18]
Four-implant over-denture (new)	1,130.07	2,199.98	3,190.67	[16-18]
Denture repair after implant failure	206.68	267.35	323.39	[16,17]
Denture repair without implant loss	65.19	84.93	103.16	[16,17]
*Material and lab cost*				
Six-implant over-denture (new)	5,070.30	5070.30	5,070.30	[18]
Four-implant over-denture (new)	4,507.82	4507.82	4,507.82	[18]
Denture repair after implant loss	160.00	160.00	160.00	HU
Denture repair without implant loss	50.00	50.00	50.00	HU

GOZ: Gebührenordnung für Zahnärzte; HU: Heidelberg University, Department of Prosthodontics dental lab.

### Measuring ‘value for money’

As it is current practice in the economic evaluation of dental care [19-27], incremental cost-effectiveness ratios were employed for comparing additional costs and additional utilities of maxillary overdentures based on six instead of four implants. Estimates of cost and health outcomes were harvested from the Markov decision tree model which was assumed to run for ten consecutive cycles, thus representing a ten-year time horizon. To incorporate uncertainties regarding model input parameters, probabilistic sensitivity analysis was implemented that assigns triangular distribution functions to failure rates and to the adjustment factor for reduced patient satisfaction in repair periods (Table 1). Monte-Carlo simulations with 50,000 repetitions were carried out to compute Cost-Effectiveness-Acceptability-Curves (CEACs) which plot the probabilities with which the treatment alternative with six implants and the four-implant solution represent preferable treatment strategies alongside differently assumed cost-effectiveness threshold levels. Accordingly, thresholds were gathered above which the preferability of the six-implant solution exceeds that of the four-implant solution. In addition to the base case scenario described above, several one-way sensitivity analyses were conducted, including alternative scenarios with respect to patient satisfaction (defined in Table 3) and with respect to the assumed parameter values for failure of dentures without implants and for patient satisfaction throughout repair periods. All data modeling and probabilistic sensitivity analyses were conducted using the software package TreeAge (TreeAge Software Inc., Williamstown, MA, USA).

**Table 3:** Alternative patient satisfaction scenarios used throughout sensitivity analysis [1: full satisfaction; 0: no satisfaction]

	**Alternative scenario A [constant satisfaction decline]**	**Alternative scenario B [proportionally increasing satisfaction decline]**
6 implants	0.89	0.89
5 implants	0.85	0.88
4 implants	0.80	0.85
3 implants	0.76	0.82
2 implants	0.72	0.77
1 implant	0.67	0.70
No implants	0.63	0.63

## Results

**Figure 2:**
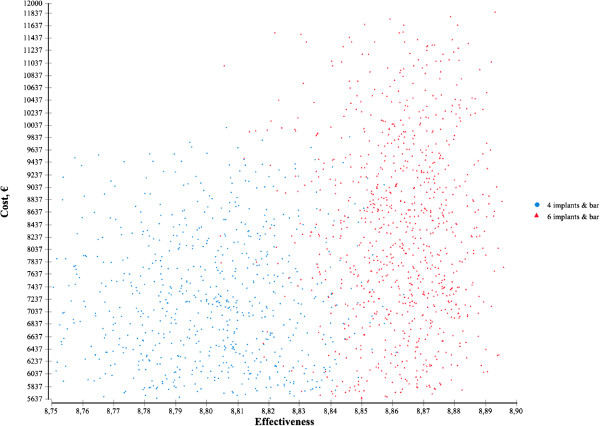
**The cost-effectiveness plane (Monte-Carlo simulation); detailed legend: Monte-Carlo simulation based on distribution functions as specified in Table**1**and on variation in treatment costs (triangular distribution function; mode corresponding to cost factor 2.3, minimum value corresponding to cost factor 1.0, maximum value corresponding to cost factor 3.5; cost calculations as shown in Table**2**).**

**Figure 3:**
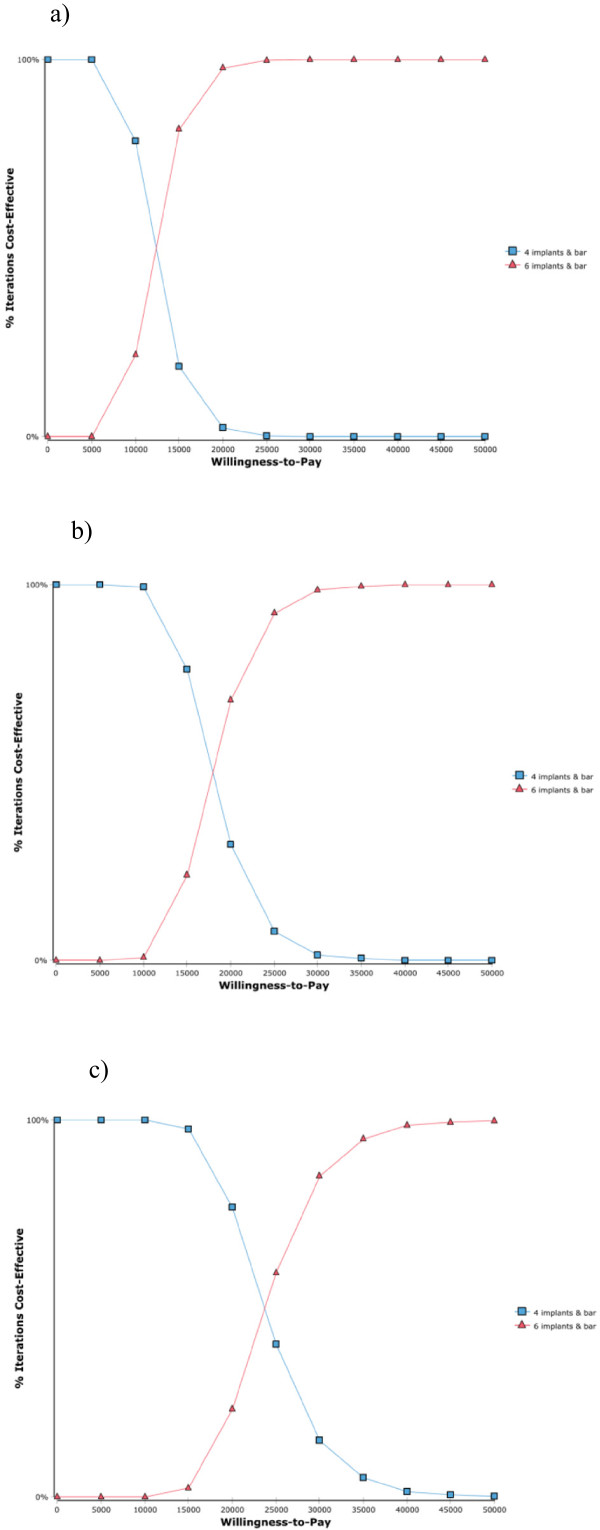
**Cost-effectiveness acceptability curves (base case scenario). a)** low treatment complexity (cost factor 1.0); **b)** average treatment complexity (cost factor 2.3); **c)** high treatment complexity (cost factor 3.5).

Figure 2 shows the cost-effectiveness scatter-plot based on Monte-Carlo simulation and incorporating potential variation in implant and denture survival rates as well as patient satisfaction in respective after-care periods (triangular distribution functions as specified in Table 1) and variation in treatment costs (triangular distribution function; mode corresponding to cost factor 2.3, minimum value corresponding to cost factor 1.0, maximum value corresponding to cost factor 3.5; cost calculations as shown in Table 2). Cost-effectiveness values achieved by the six-implant solution are represented by red triangles and cost-effectiveness values achieved by the four-implant solution are represented by blue circles. The more to the right on the plane, the higher the utility; the further up on the plane, the higher the treatment costs. Although the cost-effectiveness values for both the six-implant solution and the four-implant solution are scattered widely, there is a tendency towards the six-implant solution being located more to the right and further up on the plane than the four-implant solution. That means the six-implant solution tends to be more effective but more costly than the four-implant solution.Figure 3 shows CEACs for the base case scenario according to low treatment complexity (cost multiplication factor 1.0; Figure 3a), average treatment complexity (cost multiplication factor 2.3; Figure 3b), and high treatment complexity (cost multiplication factor 3.5; Figure 3c). All CEACs follow a similar pattern, that is for low assumed cost-effectiveness threshold levels the four-implant solution has a much higher probability of being preferable than the six-implant solution. With increasing cost-effectiveness threshold level, the probability of the four-implant solution to be preferable decreases and that of the six-implant solution increases such that the six-implant solution achieves a higher probability to be preferable after exceeding the intersection point between both CEAC curves. The respective cost-effectiveness thresholds above of which the six-implant solution achieves a higher probability to be preferable over the four-implant solution are found at 11,746 € per year of denture satisfaction gained (Figure 3a, cost factor 1.0), 17,546 € per year of denture satisfaction gained (Figure 3b, cost factor 2.3), and at 22,894 € per year of denture satisfaction gained (Figure 3c, cost factor 3.5).

Table 4 displays cost-effectiveness thresholds for the base case scenario and according to one-way sensitivity analysis based on altered assumptions about model input factors (all values are given in € per year of denture satisfaction gained). These values represent the minimum monetary amount a decision maker needs to be willing to invest per additional year of patient’s denture satisfaction in order to prefer the six-implant solution over the four-implant solution. Corresponding to the cost-effectiveness thresholds found in Figure 3 (see above), the base case scenario presents thresholds of 11,746 € (cost factor 1.0, low treatment complexity), 17,564 € (cost factor 2.3, average treatment complexity), and 22,894 € (cost factor 3.5, high treatment complexity). If assuming different scenarios of patient satisfaction as specified in Table 3, cost-effectiveness thresholds range from 863 € (alternative scenario A, cost factor 1.0) to 3,420 € (alternative scenario B, cost factor 3.5). Cost-effectiveness thresholds remained robust when the annual failure rate of dentures without implants was varied from 0.00 to 0.12. Finally, when alternating the adjustment factor for reduced patient satisfaction throughout repair periods from 0.0 (total dissatisfaction) to 1.0 (complete satisfaction), the respective cost-effectiveness thresholds ranged from 2,931 € (adjustment factor = 0.0; cost factor 1.0) to 35,040 € (adjustment factor = 1.0; cost factor = 3.5).

**Table 4:** Cost-effectiveness thresholds for preferability of six vs. four implants [in € per year of patient satisfaction]

	**Cost factor 1.0**	**Factor 2.3**	**Factor 3.5**
**Base case**	11,746 €/y*	17,564 €/y*	22,894 €/y*
Sensitivity analysis (one-way)			
*Patient satisfaction*			
Alternative scenario A (see Table 3)	863 €/y*	1,290 €/y*	1,682 €/y*
Alternative scenario B (see Table 3)	1,755 €/y*	2,624 €/y*	3,420 €/y*
*Annual failure rate of denture w/o implants*			
Failure rate = 0.00	11,746 €/y*	17,564 €/y*	22,894 €/y*
Failure rate = 0.06	11,746 €/y*	17,564 €/y*	22,894 €/y*
Failure rate = 0.12	11,746 €/y*	17,564 €/y*	22,894 €/y*
*Repair period satisfaction adjustment factor*			
Adjustment factor = 0.0	2,931 €/y*	4,382 €/y*	5,712 €/y*
Adjustment factor = 0.2	3,520 €/y*	5,263 €/y*	6,861 €/y*
Adjustment factor = 0.4	4,406 €/y*	6,588 €/y*	8,587 €/y*
Adjustment factor = 0.6	5,887 €/y*	8,803 €/y*	11,474 €/y*
Adjustment factor = 0.8	8,870 €/y*	13,263 €/y*	17,288 €/y*
Adjustment factor = 1.0	17,978 €/y*	26,882 €/y*	35,040 €/y*

*€/y: € per year of denture satisfaction gained.

## Discussion

This study is, to our knowledge, the first economic evaluation of maxillary implant overdentures so far. Given resource scarcity within and outside dental care, results from such a health economic evaluation may be highly relevant not only to patients but also to health insurers and other health care decision makers who need to decide how resources are best spent in order to increase population wellbeing. The question to address is whether relying on bar-retained maxillary overdentures based on six instead of four implants represents good value for money.

The present study found that, within a ten-year time horizon, bar-retained maxillary overdentures based on six implants provide better patient satisfaction than overdentures based on four implants but at considerably higher treatment expenses. For a base case scenario of average treatment complexity, the cost-effectiveness threshold was identified to be about 17,564 € per year of denture satisfaction gained above which the alternative with six implants is preferable over treatment including four implants. However, sensitivity analysis revealed that cost-effectiveness thresholds depend considerably on the distribution of patients’ denture satisfaction as relating to the number of implants and on the extent of satisfaction throughout denture repair periods. Plausibly, the preferability of the six-implant treatment alternative increased with more pronounced satisfaction margins in comparison with the four-implant solution. In addition, because the probability of denture and implant failure increases with decreasing number of implants, the preferability of the six-implant treatment alternative also increased with more amply constrained satisfaction throughout repair periods. In order to pinpoint the value for money of maxillary overdentures based on six instead of four implants more precisely, future research should thus specifically intend to provide more detailed insights into patient satisfaction.

Rating the value for money to society in the context of maxillary implant overdentures is further complicated by non-availability of relevant reference values, that is willingness-to-pay (WTP) per year of denture satisfaction gained. WTP can generally be defined as the maximum a person would be willing to pay for a good or a service [28], in this case for one year of satisfaction with an implant-retained maxillary overdenture. Several methods exist to measure consumer WTP [29]. These methods can be distinguished according to whether they measure consumers’ hypothetical or actual WTP, and whether they measure consumer willingness to pay directly or indirectly [30]. Methods of measuring WTP include the sealed bid auction [31], the Vickrey auction [32], conjoint analysis [33], and contingency valuation [34]. WTP investigations of oral health care are relatively rare and, so far, have focused mainly on community water fluoridation [35], orthognathic treatment [36], anaesthetic gel [37], and treatment of dentine hypersensitivity [38]. However, it seems reasonable to contemplate more generic WTP reference values which are already used by policy makers. Notably, a threshold range of £20,000 to £30,000 per quality adjusted life year (QALY) is assumed to be adopted by national health care decision makers in the United Kingdom [39], corresponding to a threshold range of about 24,000 € to 36,000 € (exchange rate as of April 10th 2014). Given that societal WTP per year of denture satisfaction may plausibly be expected to be considerably lower than societal WTP per QALY, it thus seems unlikely that a cost-effectiveness threshold of 17,564 € per year of denture satisfaction gained would imply good value for money. Nevertheless, depending on personal preferences and wealth, some patients may still favor the six-implant alternative to the four-implant solution in spite of substantially higher costs.

The present study was based on currently available evidence on clinical and patient-centered outcomes of implant- and bar-retained maxillary overdentures. We are aware that a more extensive literature search using other search engines such as EMBASE or the COCHRANE library may yield further evidence. Nevertheless, the included evidence seems highly representative of the relevant literature and can thus be considered sufficient for the purpose of the present study, i.e. to provide an economic perspective on the value for money gained through maxillary overdentures based on six as compared to four implants. The literature in this field appears to be relatively sparse and implies considerable uncertainty regarding the input parameters of our decision analytic model. In particular, the implant and denture survival rates underlying our model are based on clinical evidence with an average follow-up time of only one year and are limited to information about six and four implants only. In addition, few studies exist which provide information about patients’ satisfaction for the relevant clinical scenarios. Given that our results varied substantially with respect to simulated alterations of model input parameters, more comprehensive clinical evidence is needed in order to achieve higher accuracy within health economic evaluation. In the future, this may also better enable to model more complex clinical scenarios such as different patterns of implant loss or potential re-implantation after implant loss. In the absence of reliable evidence, we had to assume that implants are not replaced once lost but this may not fully capture existing treatment options. In view of considerable uncertainty already implied by currently available clinical evidence, we also refrained from applying discount rates to future costs and health outcomes because, generally, our results may better be understood as providing guidance for future research priorities rather than very accurate calculations of value for money to the last decimal point. Nevertheless, the present study established a suitable and widely applicable methodological framework for the economic evaluation of maxillary implant overdentures which can be applied in future calculations as well.

## Conclusion

The results of the present study suggest that bar-retained maxillary overdentures based on six implants provide better patient satisfaction than bar-retained overdentures based on four implants but are considerably more expensive. Making final judgments about value for money however requires more comprehensive clinical evidence including patient-centered health outcomes. Future clinical research should specifically examine long-term implant and denture survival as well as patient-centered outcomes of different alternatives for implant-retained overdenture treatment of the edentulous maxilla.

## Competing interests

The authors declare that they have no competing interests.

## Authors’ contributions

SL conceived the study, participated in its design and coordination, and drafted the manuscript. LF participated in the design of the study, carried out the acquisition of data, performed the health economic modeling and statistical analysis, and helped to draft the manuscript. NNG participated in the design and coordination of the study and helped to draft the manuscript. All authors read and approved the final manuscript.

## Pre-publication history

The pre-publication history for this paper can be accessed here:

http://www.biomedcentral.com/1472-6831/14/105/prepub
